# Maternal super-obesity and perinatal outcomes in Australia: a national population-based cohort study

**DOI:** 10.1186/s12884-015-0693-y

**Published:** 2015-12-02

**Authors:** Elizabeth A. Sullivan, Jan E. Dickinson, Geraldine A Vaughan, Michael J. Peek, David Ellwood, Caroline SE Homer, Marian Knight, Claire McLintock, Alex Wang, Wendy Pollock, Lisa Jackson Pulver, Zhuoyang Li, Nasrin Javid, Elizabeth Denney-Wilson, Leonie Callaway, on behalf of the Australasian Maternity Outcomes Surveillance System (AMOSS)

**Affiliations:** Faculty of Health, University of Technology Sydney, PO Box 123, Broadway NSW, 2007 Sydney, Australia; School of Women’s and Children’s Health, The University of New South Wales, Sydney, Australia; School of Women’s and Infants’ Health, The University of Western Australia, Perth, Australia; Department of Obstetrics and Gynaecology Medical School College of Medicine, Biology and Environment, The Australian National University, Canberra, Australia; Obstetrics and Gynaecology, Centenary Hospital for Women and Children, Canberra, Australia; School of Medicine, Griffith University, Queensland, Australia; Gold Coast University Hospital, Queensland, Australia; National Perinatal Epidemiology Unit, University of Oxford, Oxford, United Kingdom; Obstetrics and Gynaecology, National Women’s Health, Auckland City Hospital, Auckland, New Zealand; Judith Lumley Centre, La Trobe University, Melbourne, Australia; Department of Nursing, Melbourne School of Health Sciences, The University of Melbourne, Melbourne, Australia; Muru Marri Indigenous Health Unit, School of Public Health and Community Medicine, The University of New South Wales, Sydney, Australia; Royal Brisbane and Women’s Hospital, Brisbane, Australia; School of Medicine, The University of Queensland, Brisbane, Australia

**Keywords:** Super-obesity, Obesity, Perinatal outcomes, Pregnancy, Maternal socio-economic disadvantage, Obstetric complications

## Abstract

**Background:**

Super-obesity is associated with significantly elevated rates of obstetric complications, adverse perinatal outcomes and interventions. The purpose of this study was to determine the prevalence, risk factors, management and perinatal outcomes of super-obese women giving birth in Australia.

**Methods:**

A national population-based cohort study. Super-obese pregnant women (body mass index (BMI) >50 kg/m^2^ or weight >140 kg) who gave birth between January 1 and October 31, 2010 and a comparison cohort were identified using the Australasian Maternity Outcomes Surveillance System (AMOSS). Outcomes included maternal and perinatal morbidity and mortality. Prevalence estimates calculated with 95 % confidence intervals (CIs). Adjusted odds ratios (ORs) were calculated using multivariable logistic regression.

**Results:**

370 super-obese women with a median BMI of 52.8 kg/m^2^ (range 40.9–79.9 kg/m^2^) and prevalence of 2.1 per 1 000 women giving birth (95 % CI: 1.96–2.40). Super-obese women were significantly more likely to be public patients (96.2 %), smoke (23.8 %) and be socio-economically disadvantaged (36.2 %). Compared with other women, super-obese women had a significantly higher risk for obstetric (adjusted odds ratio (AOR) 2.42, 95 % CI: 1.77–3.29) and medical (AOR: 2.89, 95 % CI: 2.64–4.11) complications during pregnancy, birth by caesarean section (51.6 %) and admission to special care (HDU/ICU) (6.2 %). The 372 babies born to 365 super-obese women with outcomes known had significantly higher rates of birthweight ≥4500 g (AOR 19.94, 95 % CI: 6.81–58.36), hospital transfer (AOR 3.81, 95 % CI: 1.93–7.55) and admission to Neonatal Intensive Care Unit (NICU) (AOR 1.83, 95 % CI: 1.27–2.65) compared to babies of the comparison group, but not prematurity (10.5 % versus 9.2 %) or perinatal mortality (11.0 (95 % CI: 4.3–28.0) versus 6.6 (95 % CI: 2.6- 16.8) per 1 000 singleton births).

**Conclusions:**

Super-obesity in pregnancy in Australia is associated with increased rates of pregnancy and birth complications, and with social disadvantage. There is an urgent need to further address risk factors leading to super-obesity among pregnant women and for maternity services to better address pre-pregnancy and pregnancy care to reduce associated inequalities in perinatal outcomes.

## Background

The prevalence of obesity (body mass index (BMI) ≥ 30 kg/m^2^) among women of reproductive age continues to rise in developed countries, with Australia at 28.3 % among the highest in the world ahead of United Kingdom [1] and similar to the United States. It is estimated that about one in five women giving birth in Australia are obese [2]. Of increasing concern is the rising rate of so-called super-obesity, defined as a BMI of ≥ 50 kg/m^2^ in pregnancy, or women weighing 225 % of ideal body weight [3, 4]. Super-obesity is associated with significantly elevated rates of obstetric complications, adverse perinatal outcomes and interventions including pre-eclampsia, gestational diabetes mellitus (GDM), preterm birth, caesarean section, general anaesthesia, wound infection, intensive care admission, macrosomia, neonatal hypoglycaemia and congenital anomalies [3–6]. However, there has been no national study of super-obesity in Australian women giving birth. A population study from the United Kingdom reported the prevalence of women with BMI ≥50 kg/m2 as 8.7 per 10 000 women giving birth or 0.1 % [7]. In contrast, the prevalence of super-obesity ranged from 1.8 % [4] in a 7-year (2000 to 2006) US retrospective cohort study in Missouri, to 2.2 % for a 12-year (1996 to 2007) case series of 19 700 women giving birth in South Carolina [5]. A retrospective 12-year cohort study of 75 432 women giving birth in a Brisbane hospital (Australia) found a significant increase in the proportion of Class III obesity (≥40 kg/m2) during the course of the study [8]. This suggests that the prevalence of super-obesity among women giving birth is also on the rise in Australia although this has not been previously reported [8].

The objective of this study was to determine the prevalence, risk factors, management and perinatal outcomes of super-obese women giving birth in Australia; and to determine the effect of maternal super-obesity on perinatal outcomes compared with other women. We hypothesized that pregnancy in super-obese women compared with other women is associated with a higher risk of maternal morbidity and adverse perinatal outcomes.

## Methods

### Study design and population

A national, prospective cohort study was undertaken using the Australasian Maternity Outcomes Surveillance System (AMOSS). The AMOSS methods have been described in detail elsewhere [9]. Women were identified by participating AMOSS sites, responding to a monthly email that included negative reporting of whether a case had been identified between January and October 2010. Hospital (*n* = 226) sites progressively joined AMOSS on completion of relevant ethics/governance processes and were included for the period they participated in the study. The denominator of 171 289 women giving birth was calculated using the number of days of participation in the study multiplied by number of births per day for that hospital and gave approximate coverage of 66 % of all women giving birth in Australia. The case definition included any pregnant woman of 20 weeks’ gestation or more who, at any point in pregnancy, had a BMI of greater than 50 kg/m^2^ or a weight of more than 140 kg. The case definition was clinician informed with a weight of >140 kg at any point in pregnancy considered super-obese irrespective of having a BMI <50 kg/m^2^. The comparison group for a series of AMOSS studies were the two women who gave birth immediately before women with placenta accreta and/or women who underwent a peripartum hysterectomy between January 2010 and December 2011 [10]. The comparison group represented the general population of women giving birth in Australia and New Zealand and inclusion criteria did not include BMI, however all comparison women had a BMI ≤50 kg/m^2^ or weight ≤140 kg*.*

A questionnaire completed by AMOSS site coordinators for all eligible women sought information on demographic and pregnancy factors, obstetric interventions and perinatal outcomes as well as models of antenatal care, specified medical and obstetric complications and bariatric equipment (e.g., high-weight capacity bed, operating table, hoist, chair) availability. Free-text responses to questions regarding medical/obstetric morbidity were categorised according to ICD-10 AM codes.

We anticipated identifying 264 super-obese women and 528 comparison women over 12 months, based on the prevalence of the United Kingdom study of 8.7 per 10 000 women giving birth [7]. These numbers give a power of 80 % at the 5 % level of significance to detect difference in proportions of outcomes (gestational diabetes, caesarean section and admission to NICU) by 10 % in study group over a range of incidences from 5 to 30 % in the comparison group.

#### Other study factors

The woman’s age was calculated in completed years at the time of the antenatal care booking visit and classified into four categories: <25, 25–29, 30–34, and ≥ 35 years. Other demographic characteristics such as parity (0, 1–2, and ≥ 3), Indigenous status, marital status, admission as private/public patient, smoking during pregnancy, socio-economic status (Australian socio-economic indices for areas (SEIFA) of relative advantage/disadvantage quintile) [11], previous caesarean section, multiple pregnancy and assisted reproductive technology treatment were recorded.

### Outcomes

Models of care, obstetric interventions, and birth outcomes were measured for both groups. Health professional involvement during antenatal care, specific medical and obstetric complications and bariatric equipment availability were recorded for the super-obese women only.

### Statistical analysis

Prevalence estimates with 95 % confidence intervals (CIs) were calculated. Distribution of BMI was graphically compared between super-obese women and the comparison group. Chi-square or Fisher’s exact test was used to investigate difference in obstetric interventions and birth outcomes of study and comparison groups. Multivariable logistic regression was used to examine the medical and obstetric complications (gestational diabetes, gestational hypertension, pre-eclampsia, etc.), labour characteristics (onset of labour and method of delivery), maternal outcomes (admission to ICU or HDU) and perinatal outcomes (birthweight ≥4 500 g, admission to NICU, and need for transfer). Odds ratio (OR) and adjusted odds ratio (AOR) and 95 % confidence interval (CI) were calculated. Adjustment was made for maternal age, maternal Indigenous status, marital status, admission as private/public patient, smoking status, assisted reproductive technology, parity, multiple gestation and socio-economic status. Any p-values less than 0.05 were considered statistically significant. Data were analysed using the Statistical Package for the Social Sciences software, version 22.0 (IBM Corporation, Somers, NY, USA).

### Ethics approval

Ethics approval for AMOSS was granted by NSW Population and Health Services Research Ethics Committee and multiple Human Research Ethics Committees across Australia [12] and the multiregional ethics approval in New Zealand. The AMOSS studies are considered low-risk under (Australian) National Health and Medical Research Council (NHMRC) guidelines. The data collected from case notes by onsite AMOSS data coordinators were de-identified, and no consent was required by participants. Data were reported at an aggregate level only [12].

## Results

**Fig. 1:**
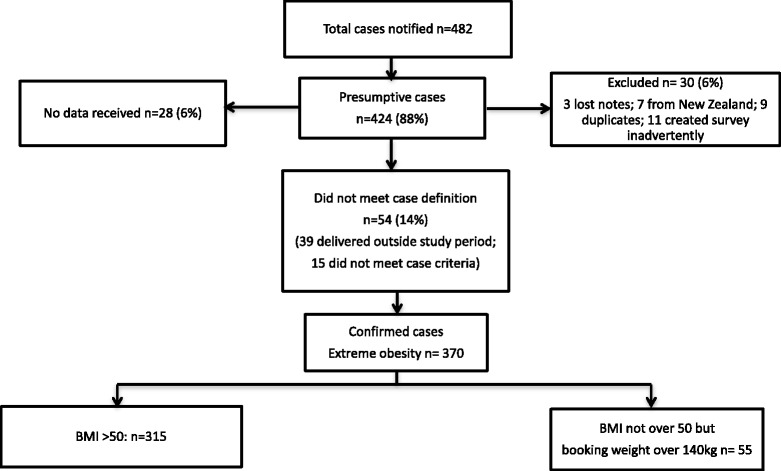
Surveillance and confirmed cases of super-obese women who gave birth in Australia, 2010

**Fig. 2:**
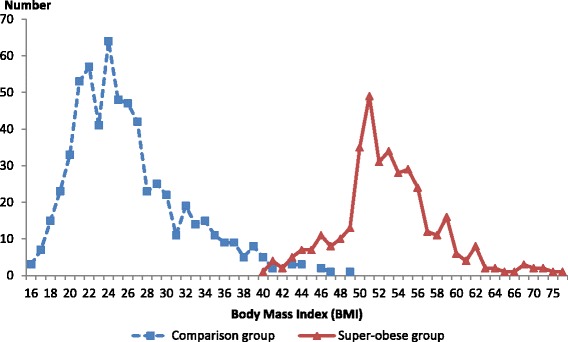
Distribution of body mass index of super-obese and comparison women who gave birth in Australia, 2010

A total of 370 super-obese pregnant women (297 women had a BMI of greater than 50 kg/m^2^ and 73 women had a weight of more than 140 kg) were confirmed as cases with an estimated prevalence of 2.14 per 1000 (95 % CI: 1.96–2.40) women giving birth (Fig. 1). Data were available for 621 women in the comparative group. The median BMI (Fig. 2) of the super-obese women was 52.8 kg/m^2^ (range, 40.9–79.9 kg/m^2^) compared to 24.8 kg/m^2^ (range, 16.3–48.9 kg/m^2^) for comparison women. The median weight of super-obese women was 156 kg (range 108–204 kg) which was over twice the median weight for the comparison women of 67 kg (range 42–138 kg). Demographic and pregnancy-related characteristics of super-obese and comparison groups are shown in Table 1. The super-obese women (cases) were of similar age but of significantly higher parity than the comparison women (parity ≥ 3: 22.2 % versus 9.2 %, *p* < 0.01). They were more likely to smoke (23.8 % versus 16.1 %, *p* < 0.01), to be socio-economically disadvantaged (lowest SEIFA quintile: 17.8 % versus 7.9 %, *p* < 0.01) and be admitted as a public patient (96.2 % versus 76.7 %, *p* < 0.01).

**Table 1:** Demographic and obstetric characteristics among super-obese and comparison women who gave birth in Australia, 2010

	Super-obese group (*N* = 370)		Comparison (*N* = 621)		*P* value
	No.	%	No.	%	
Age (years)					
< 25	60	16.2	99	15.9	1.00
25–29	109	29.5	185	29.8	
30–34	110	29.7	187	30.1	
≥ 35	91	24.6	150	24.2	
Indigenous status					
No	341	92.2	572	92.1	0.23
Yes	17	4.6	19	3.1	
Not stated	12	3.2	30	4.8	
Marital status					
Single	71	19.2	71	11.4	<0.01
Married/cohabit	277	74.9	508	81.8	
Not stated	22	5.9	42	6.8	
Private health insurance					
No	356	96.2	474	76.3	<0.01
Yes	14	3.8	145	23.3	
Not stated	0	0.0	2	0.3	
Smoking during pregnancy					
No	265	71.6	484	77.9	<0.01
Yes	88	23.8	100	16.1	
Not stated	17	4.6	37	6.0	
Assisted reproductive technology					
No	357	96.5	574	92.4	0.17
Yes	11	3.0	29	4.7	
Not stated	2	0.5	18	2.9	
Parity					
0	115	31.1	251	40.4	<0.01
1–2	173	46.8	313	50.4	
3+	82	22.2	57	9.2	
Multiple gestation pregnancy					
No	362	97.8	608	97.9	0.81
Yes	8	2.2	12	1.9	
Not stated	0	0.0	1	0.2	
Socio-economic status^a^					
Most disadvantage 1	66	17.8	49	7.9	<0.01
2	68	18.4	73	11.8	
3	109	29.5	121	19.5	
4	80	21.6	181	29.1	
Least disadvantage 5	40	10.8	191	30.8	
Not stated	7	1.9	6	1.0	
Previous caesarean section (exclude primiparous)					
No	140	54.9	250	67.6	<0.01
Yes	112	43.9	115	31.1	
Not stated	3	1.2	5	1.4	

^a^Socio-Economic Indexes for Areas Index for Relative Socio-economic Disadvantage quintiles assigned to those residents in the most disadvantaged areas to Quintile 1 and those in the least disadvantaged areas to Quintile 5

### Models of care

The majority of super-obese women had a hospital-based, medical model of care with few under the care of midwives or private obstetricians, which was significantly different from the comparison group (Table 2). Changes in the model of care and rates of transfer between hospitals were higher for the super-obese group compared with comparison women (Table 2). Fewer than half (*n* = 173, 46.8 %) of the super-obese women saw a dietician during pregnancy, while 70.5 % (*n* = 261) consulted obstetric anaesthetists and 17.8 % (*n* = 66) consulted maternal-fetal medicine specialists during pregnancy (data unavailable for comparison group). Multi-disciplinary meetings were held for 12 % of super-obese women during the antenatal period to plan management.

**Table 2:** Model of care among super-obese and comparison women who gave birth in Australia, 2010

	Super-obese group (*N* = 370)		Comparison (*N* = 621)		*P* value
	No.	%	No.	%	
Lead care provider					
General practitioner	72	19.5	122	19.6	<0.01
Hospital medical	247	66.8	175	28.2	
Hospital midwife	34	9.2	182	29.3	
Private obstetrician	17	4.6	140	22.5	
Not stated	0	0.0	2	0.3	
Changed during pregnancy					
No	312	84.3	566	91.1	<0.01
Yes	56	15.1	55	8.9	
Not stated	2	0.5	0	0.0	
Transfer					
No	326	88.1	596	96.0	<0.01
Yes	44	11.9	25	4.0	
Timing of maternal transfer					
Antepartum	34	77.3	19	76.0	0.77
Intrapartum/Postpartum	9	20.5	6	24.0	
Not stated	1	2.3	1	0.0	

### Medical and obstetric complications during pregnancy

Super-obese women had significantly higher rates of obstetric (42.0 % versus 23.2 %; AOR: 2.42, 95 % CI: 1.77–3.29) and medical (33.3 % versus 13.0 %; AOR: 2.89, 95 % CI: 2.64–4.11) complications during pregnancy (Table 3). Super-obese women were significantly more likely to develop gestational diabetes (15.6 % versus 7.2 %; AOR: 2.52, 95 % CI: 1.58–4.65), pre-eclampsia (8.5 % versus 2.6 %; AOR: 3.43, 95 % CI: 1.72–6.84) or gestational hypertension (12.3 % versus 1.5 %; AOR: 10.24, 95 % CI: 4.67–22.44) than comparison women. Of the 18 (4.9 %) of super-obese women who had antenatal thromboprophylaxis, nine (2.4 %) were given low-molecular weight heparin.

**Table 3:** Labour and birth characteristics among super-obese and comparison women who gave birth in Australia, 2010

	Super-obese group (*N* = 370)		Comparison (*N* = 621)		*P* value
	No.	%	No.	%	
Multiple births					
Singleton	362	97.8	608	97.9	0.81
Twin	8	2.2	12	1.9	
Not stated	0	0.0	1	0.2	
Labour					
No	125	33.8	139	22.4	<0.01
Yes	241	65.1	482	77.6	
Not stated	4	1.1	0	0.0	
Induction of labour					
No	100	41.5	351	72.8	<0.01
Yes	140	58.1	129	26.8	
Not stated	1	0.4	2	0.4	
Method of birth					
Vaginal birth	176	47.6	424	68.3	<0.01
Caesarean section	191	51.6	197	31.7	
Not stated	3	0.8	0	0.0	
Caesarean section					
Planned	103	53.9	122	61.9	0.1
Unplanned	87	45.5	73	37.1	
Not stated	1	0.5	2	1.0	
Use of general anaesthetic					
No	172	90.1	191	97.0	0.01
Yes	19	9.9	6	3.0	

### Labour and birth

Compared to the comparison women, the super-obese women were more likely to undergo induction of labour or no labour (Table 4). The likelihood of having caesarean section was significantly higher among super-obese women (51.6 %) than the comparison women (31.7 %) (AOR: 2.73, 95 % CI: 2.02–3.69) with 9.9 % of caesarean section performed under general anaesthesia (Table 3). The most common indications for planned caesarean section were previous CS (72 %, *n* = 76), abnormal fetal presentation (breech, transverse, or unstable lie (18 %, *n* = 19), macrosomia (7 %, *n* = 7) and maternal medical complications (7.5 %, *n* = 8). Shoulder dystocia occurred in 11 (6 %) of super-obese women who gave birth vaginally.

**Table 4:** Maternal, obstetric and perinatal outcomes among super-obese and comparison women who gave birth in Australia, 2010^a^

	Super-obese group	Comparison	OR	AOR^b^
	(*N* = 370)	(*N* = 621)		
	%	%		
Complications during pregnancy				
Medical problems during pregnancy	33.3	13	3.35 (2.43–4.62)	2.89 (2.64–4.11)
Obstetric problems during pregnancy	42	23.2	2.40 (1.81–3.17)	2.42 (1.77–3.29)
Gestational diabetes	15.6	7.2	2.36 (1.56–3.59)	2.52 (1.58–4.65)
Gestational hypertension	12.3	1.5	9.33 (4.50–19.33)	10.24 (4.67–22.44)
Preeclampsia	8.5	2.6	3.42 (1.85–6.35)	3.43 (1.72–6.84)
Obstetric				
Labour	65.8	77.6	0.55 (0.42–0.74)	0.49 (0.35–0.68)
Induction of labour	58.3	26.9	3.81 (2.75–5.28)	4.33 (3.21–6.24)
Caesarean section	52	31.7	2.33 (1.79–3.65)	2.73 (2.02–3.69)
Perinatal outcomes (singletons only)				
Birthweight ≥4500 g	10.1	0.8	13.44 (5.22–34.57)	19.94 (6.81–58.36)
Admitted to NICU	23.7	13.9	1.93 (1.38–2.71)	1.83 (1.27–2.65)
Need for transfer	8.5	2.7	3.39 (1.82–6.31)	3.81 (1.93–7.55)
Preterm birth <37 weeks	10.1	8.1	1.28 (0.81–2.00)	1.18 (0.72,1.93)
Maternal outcomes				
Admission to ICU	2.2	0.5	4.56 (1.25–17.32)	7.38 (1.52–35.87)
Admission to HDU	4.3	0.8	5.58 (2.43–15.37)	5.40 (1.78–16.38)
Admission to either ICU or HDU	6.2	1.3	5.09 (2.25–11.51)	5.67 (2.31–13.93)

*OR* odds ratio, *AOR* adjusted odds ratio, *NICU* neonatal intensive care unit, *ICU* intensive care unit, *HDU* high dependency unit

^a^Table 4 data excludes not stated and this may produce discrepant results to previous tables where not stated is included

^b^Outcomes are adjusted for age, Indigenous status, marital status, private health insurance, smoking during pregnancy, assisted reproductive technology, parity, multiple gestation pregnancy, and Socio-Economic Indexes for Areas Index for Relative Socio-economic Disadvantage

### Perinatal outcomes

Birth outcomes were known for 365 (98.6 %) of the super-obese women (*n* = 372 infants, including 7 sets of twins. Super-obese women were significantly more likely to give birth to babies with a birthweight ≥4 500 g (AOR: 19.94, 95 % CI: 6.81–58.36) (Table 4). Thirty-six (9.8 %) of 362 singleton infants born to super-obese women had a birthweight ≥4 500 g, in contrast to 0.8 % (*n* = 5/608) of the singleton infants in the comparison group. Of the 36 singletons with a birthweight ≥4 500 g, 16.6 % of their mothers had pre-existing diabetes. There was no significant difference in preterm birth between super-obese and comparison women (*n* = 39/370, 10.5 % versus *n* = 57/621, 9.2 %) (Table 4). Of the singletons born to super-obese women, 22.9 % (*n* = 83/362) were admitted to the neonatal intensive care unit (NICU) compared to 13.7 % (*n* = 83/608) of singletons in the comparison group (Table 4). The perinatal mortality rate for the infants born to super-obese women was 11.0 per 1 000 (95 % CI: 4.3–28.0) singleton births which consisted of four stillbirths (one <30 weeks) and no neonatal deaths. There were three stillbirths (2 antepartum and 1 intrapartum) among the infants born in the comparison group (all <30 weeks) and one neonatal death, giving a perinatal mortality rate of 6.6 per 1 000 (95 % CI: 2.6, 16.8) singleton births. Three of the four super-obese women who had stillbirths reported gestational hypertension compared to none of the comparison women.

### Postpartum maternal complications

Thirteen per cent (49/370) of the super-obese women experienced a postpartum infection; of those, 69 % (*n* = 34) had undergone a caesarean section. The most common infection was wound infection (*n* = 25, 51 %) with 12 (24 %) women having multiple complications from infection. Postnatally, 224 (60.5 %) super-obese women received thromboprophylaxis, of these, 78.1 % received low-molecular-weight heparin (LMWH). Postnatal thromboprophylaxis was administered in 29.7 % of super-obese women who gave birth vaginally compared to 90.5 % of super-obese women following caesarean section (*p* < 0.01). Of the 18 women who did not receive thromboprophylaxis following caesarean section, two women were aged >35 years, six continued to smoke during pregnancy and five had a history of hypertensive disorders (HTD). The median length of stay for super-obese women was 4 days (range 1–32 days). Super-obese women were significantly more likely to be admitted to a High Dependency Unit (HDU) or Intensive Care Unit (ICU) (6.2 % versus 1.3 %; AOR: 5.67, 95 % CI: 2.31–13.93) compared to the comparison women (Table 4). No maternal deaths were woman reported in either group.

## Discussion

Super-obesity was reported in more than 1 in 500 women giving birth in Australia with these women experiencing higher rates of obstetric complications and adverse perinatal outcomes. Compared to other women, the birth experience of super-obese women was characterised by higher rates of caesarean section, general anaesthesia, admission to HDU and ICU and hospital transfer. Fewer than one in 10 super-obese women accessed a midwifery led model of care or private obstetric care with the usual model of care being a hospital-based, medical model. Super-obese women had more than twice the risk of caesarean section (CS) with almost half of the CS unplanned and around 10 % conducted under general anaesthesia. Maternal obesity may be an independent risk factor for CS as it interferes with the progress of labour, specifically the arrest of dilation in active phase labour [13]. The higher rate of CS has been explained in other studies by the association of super-obesity with conditions such as gestational diabetes, gestational hypertension and preeclampsia [14–16], all of which were more prevalent in women in our study. This is consistent with other research including comparison with other obese women (BMI 30.0–49.9 kg/m^2^) [5]. Other studies of super-obese women (BMI ≥ 50 kg/m^2^) have reported higher rates of both early preeclampsia (AOR: 2.97, 95 % CI: 2.07- 4.26) and late preeclampsia (AOR: 4.79, 95 % CI: 4.26–5.39) compared with normal weight mothers (BMI = 18.5–24.9 kg/m^2^) [6]. While clinicians have raised concerns about ability to monitor progress of labour and assess fetal wellbeing in obese women [17], it is argued that there is insufficient evidence to justify a routine policy of CS for all super-obese women solely because of higher recorded rates of complications, but that the mode of birth should be based on a careful assessment of risk factors [18]. Hospital guidelines and other recommendations would suggest thromboprophylaxis for women in the study group who had caesarean sections is warranted. There is, however, little or no differentiation of risk according to degree of obesity [19–21]; although Martin et al. highlights the dose–response of increased risk of thromboembolic events and eclampsia as BMI increases and suggests this can be usefully applied to other pregnancy-related complications [16]. This amplified risk of morbidity according to BMI is consistent with the Mbah study discussed earlier and showed a significantly increased risk of pre-eclampsia between super-obese women (BMI ≥ 50 kg/m^2^ adjusted odds ratio 4.71 [4.20–5.28]) compared to women with Class III obesity (3.75 [3.59–3.92] [6].

The number of infants with birth weight > 4500 g found in our cohort is similar to the UKOSS study of super-obese women that reported a rate of almost nine percent [7]. The long term clinical impact of a high birthweight is unknown. While some studies [22] suggest these infants may have higher rates of obesity and metabolic syndrome in adolescence and adulthood, whether this is a product of the intrauterine environment or of growing up in an environment where children are exposed to sociodemographic factors that promote the development of obesity is uncertain. There were elevated rates of admission of infants to special and intensive care units placing a higher burden on the health system. A perinatal mortality rate of 11.0 per 1000 was not different to that in the comparison group or to national Australian data [23] and was slightly lower than that reported in the UKOSS study on super-obese women (16.0 per 1000) [7]. There was no difference in the rate of preterm birth among super-obese group, so any difference in perinatal outcomes was not related to prematurity.

The rising prevalence of obesity in Australian women of reproductive age suggests that strategies of weight loss, diet, exercise and bariatric surgery have been of limited benefit or that women are not aware of the potential health adverse outcomes associated with obesity in pregnancy. Our study finds that 2.1 per 1000 women giving birth in Australia are super-obese. The Royal Australia and New Zealand College of Obstetricians and Gynaecologists (RANZCOG) recommends pre-conception management of obesity and weight loss through lifestyle approaches of exercise and nutrition, bariatric surgery, nutritional supplementation and psychosocial support. It also recommends that women should have their BMI measures at their first antenatal consultation and, if indicated, multidisciplinary care should be organised to advise and monitor about gestational weight gain, nutritional supplementation, exercise and ensure access to antenatal facilities with appropriate equipment [24].

The National Institute for Health and Clinical Excellence has recommended bariatric surgery as a first-line option for adults with BMI > 50 kg/m^2^, instead of lifestyle interventions or drug treatment [25]. Studies have shown that super-obese women who had bariatric surgery prior to pregnancy had lower rates of gestational hypertension, pre-eclampsia, gestational diabetes, and macrosomic infants compared with super-obese women who had not had surgery [26–28]. Nutritional deficiencies during pregnancy have been reported among women following bariatric surgery [29] and nutritional follow-up and careful weight gain management should be provided. A recent systematic review suggests that antenatal interventions targeting diet and/or physical activity have mixed results [30]. Some studies show a reduction in maternal weight gain from antenatal dietary intervention but no effect on maternal or infant morbidity. This may reflect the limitation of intervention being focused on dietary advice alone rather than a more holistic approach which provides support for the woman and her general wellbeing. There are little data on interventions in super obese women. Interventions in the postnatal period should be considered to encourage postpartum weight loss and improve outcomes in subsequent pregnancies [31].

A strength of this study was the use of a comparison group that was representative of the women giving birth in Australia [23]. This allowed investigation of whether there was an increased clinical impact and associated burden on health services for women with super obesity. Martin et al. [16] suggest the need for differential management of super-obese pregnant women compared to the lower range of BMI 40–49.9 kg/m^2^, including the need for larger antibiotic dosage for women with BMIs of >50 kg/m^2^ to that of women with BMI of >35 kg/m^2^, and a need for more research on the specific anticoagulation requirements according to tiered classification of obesity [5, 16]. A limitation of this approach is that it potentially decreases the capacity to detect difference in our study between the super-obese group and the comparison group in maternal and perinatal outcomes due to the inclusion of obese women in the comparison group.

A limitation of the study was that there was no specific question on whether the super-obese women had previous bariatric surgery. There was a question on previous abdominal surgery with a free text response possible. Interestingly, nine super-obese women and one control reported gastric banding suggesting that any further research on super-obese women should include questions on bariatric surgery and laparoscopic-bands. Pre conception options for super-obese women may be the insertion of an adjustable laparoscopic band or gastric sleeve procedure prior to pregnancy. These procedures are associated with fewer nutritional deficiencies than bariatric surgery, and several observational studies have reported encouraging results, including lower incidence of gestational diabetes, pregnancy induced hypertension and lower weight gain during pregnancy. However women with adjustable laparoscopic bands require close monitoring from a multidisciplinary team and may need the band adjusted during pregnancy, especially in women with frequent vomiting [32]. Adjustable laparoscopic band surgery and or gastric sleeves are rarely available in the public or Medicare funded hospital system in Australia. In our sample of super obese women, only 3.8 % had private health insurance, and over one third were from the two least advantaged quintiles of socio-economic status, suggesting that lap band surgery pre-pregnancy may not be an option for many women.

Measurement error of BMI is another potential limitation of the study. Despite recommendations for maternal BMI to be recorded at the booking visit [24, 33] this was not done at all participating AMOSS sites nor were serial measures of BMI throughout pregnancy available. In a separate survey to participating sites in 2010, BMI at booking was routinely undertaken at 74 % of the 195 sites that responded. There may be some information bias regarding the BMI as weight and/or height could be self-reported or measured depending upon the maternity unit practice. At this level of obesity, some error in the BMI precision is likely to have minimal impact on the generalisability of the results, as alternative measures such as skinfolds are impractical and unreliable in general clinical practice.

A potential limitation of the study was the incremental participation of maternity sites in AMOSS over the course of the study which may have impacted recruitment of cases. Conversely, a strength of the study is that the findings are consistent with routine perinatal data which also demonstrates variation in the prevalence of super-obesity across jurisdictions ranging from 1.8 per 1000 births in NSW [34] to 3.67 per 1000 in Queensland [35] and 4.68 per 1000 births in Western Australia [36]. A second strength was the distribution of participating sites which was representative of Australian maternity services [37].

The increasing prevalence of super-obesity has important implications for maternity services as the evidence suggests current strategies have had limited impact. Super-obesity is associated with and may be a manifestation of complex socio-economic disadvantage and needs innovative interventions and strategies to address underlying health inequity. This study found over a third of super-obese women were in the two most disadvantaged quintiles with only 10.8 % in the least disadvantaged and confirms previous research findings of lower socio-economic status being associated with super-obesity [38–41]. Super obese women risk substantial co-morbidity affecting both them and their offspring. This calls for targeted strategies to address weight gain before, within and between pregnancies, that are appropriate, collaborative and provide training for clinicians within the health services. In the absence of effective interventions to enable women to lose weight (or maintain weight) during pregnancy, super-obese women planning a pregnancy should be supported to make lifestyle changes or consider laparoscopic band, gastric sleeve or bariatric surgery prior to pregnancy.

## Conclusions

The findings from our study underline the imperative to prioritise initiatives that address the increased perinatal risks of super-obese women and their babies. The overall resource burden of maternity care for super-obese women was evident with higher rates of obstetric and medical complications, intervention in pregnancy and childbirth, and for infants postnatally. Super-obesity in pregnancy is associated with social disadvantage. There is an urgent need to address pre-pregnancy and pregnancy care and ensure that appropriate initiatives are in place to reduce associated inequalities in perinatal outcomes and future pregnancies of super-obese women.
